# Difficult intubation in an asymptomatic patient with tracheobronchopathia osteochondroplastica

**DOI:** 10.1002/rcr2.526

**Published:** 2020-02-03

**Authors:** Dona Kafili, Timothy Sampson, Sean Tolhurst

**Affiliations:** ^1^ Department of Respiratory Medicine Greenslopes Private Hospital Greenslopes Queensland Australia; ^2^ Department of Anaesthesiology Greenslopes Private Hospital Greenslopes Queensland Australia

**Keywords:** Bronchoscopy, difficult intubation, preoperative assessment, tracheobronchopathia osteochondroplastica

## Abstract

Tracheobronchopathia osteochondroplastica (TO) is a rare, benign, slowly progressive disease of unknown aetiology. It is characterized by numerous sessile, cartilaginous, or osseous submucosal nodules distributed throughout the anterolateral walls of the trachea and projecting into the laryngotracheobronchial lumen. The diagnosis is usually incidental with distinct bronchoscopic or computed tomography (CT) scan findings. We have identified a case of asymptomatic TO through fibreoptic bronchoscopy and biopsy after having a difficult intubation for elective prostate surgery.

## Graphical abstract

Tracheobronchopathia osteochondroplastica (TO) is a rare, benign, slowly progressive disease of unknown aetiology. It is characterized by numerous sessile, cartilaginous, or osseous submucosal nodules distributed throughout the anterolateral walls of the trachea and projecting into the laryngotracheobronchial lumen. The diagnosis is usually incidental with distinct bronchoscopic or computed tomography (CT) scan findings. We present a case of asymptomatic TO identified through fibreoptic bronchoscopy and biopsy after having a difficult intubation for elective prostate surgery.

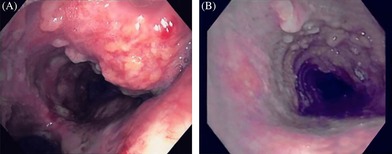

## Introduction

Tracheobronchopathia osteochondroplastica (TO) is a rare, benign, slowly progressive disease of unknown aetiology. It is characterized by numerous sessile, cartilaginous, or osseous submucosal nodules distributed throughout the anterolateral walls of the trachea and projecting into the laryngotracheobronchial lumen. TO generally occurs between the fourth and the seventh decades of life without gender predominance 1, 2, 3.

Manifestation of this condition significantly varies from asymptomatic to non‐specific respiratory symptoms including chronic cough, dyspnoea, recurrent pneumonia, and difficult intubation 4. Diagnosis is usually through characteristic radiological and bronchoscopic findings. Although biopsy is not essential, it helps exclude other differentials such as human papilloma virus (HPV) infection, amyloidosis, sarcoidosis, or relapsing polychondritis 5.

This case report illustrates a patient with asymptomatic TO that was identified following difficult intubation for an elective procedure.

## Case Report

A 73‐year‐old retired pilot was admitted for an elective radical prostatectomy for Gleason 9/10 prostate cancer. His past history includes previous coronary artery bypass graft (CABG) in 1992 which was uneventful. He is an ex‐smoker with a 5 pack‐year history and currently lives on a cattle farm.

Prior to surgery, his perioperative assessment did not identify any potential airway concerns. He underwent uneventful intravenous induction of general anaesthesia with easy bag mask ventilation. Laryngeal view was Cormack and Lehane Grade II, but it was difficult to pass a size 8 endotracheal tube (ETT). Eventually, a size 6 ETT was passed with assistance from a bougie and a Concurrent Media Access Control (C‐MAC) video laryngoscope. Position of the tube was confirmed with fibreoptic bronchoscopy during which sessile lesions were seen on the lateral wall of the trachea. Surgery proceeded as there was no difficulty with ventilation.

After recovering from his prostatectomy, he was referred to the respiratory team for further assessment. Review of previous prostate‐specific membrane antigen (PSMA) positron emission tomography (PET), completed prior to his prostate surgery, identified calcified lesions in trachea and right main bronchus. These findings were not listed on the initial report. Subsequent fibreoptic bronchoscopy revealed normal vocal cords with multiple mucosal lesions, throughout the airways, especially in the trachea, right main bronchus, and right bronchus intermedius. These were associated with up to 40% narrowing of the tracheal lumen.

Histological analysis of endobronchial cryoprobe biopsies confirmed TO, evidenced by squamous metaplasia of epithelium with prominent degrees of metaplastic trabeculated bone within submucosa.

**Figure 1:**
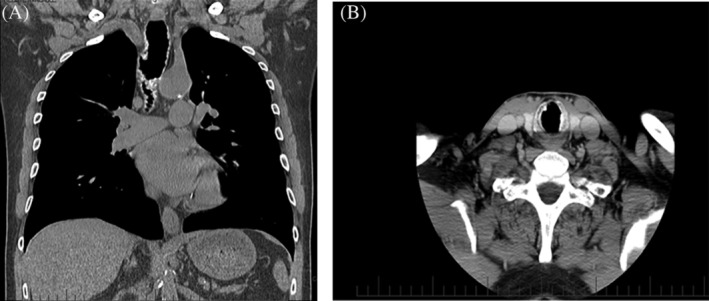
Irregular thickening of anterolateral wall of tracheal with calcification. (A) Coronal view shows the protrusion of osteocartilaginous nodules, causing narrowing of the tracheal lumen. (B) Transverse view shows that the posterior wall of trachea is spared.

**Figure 2:**
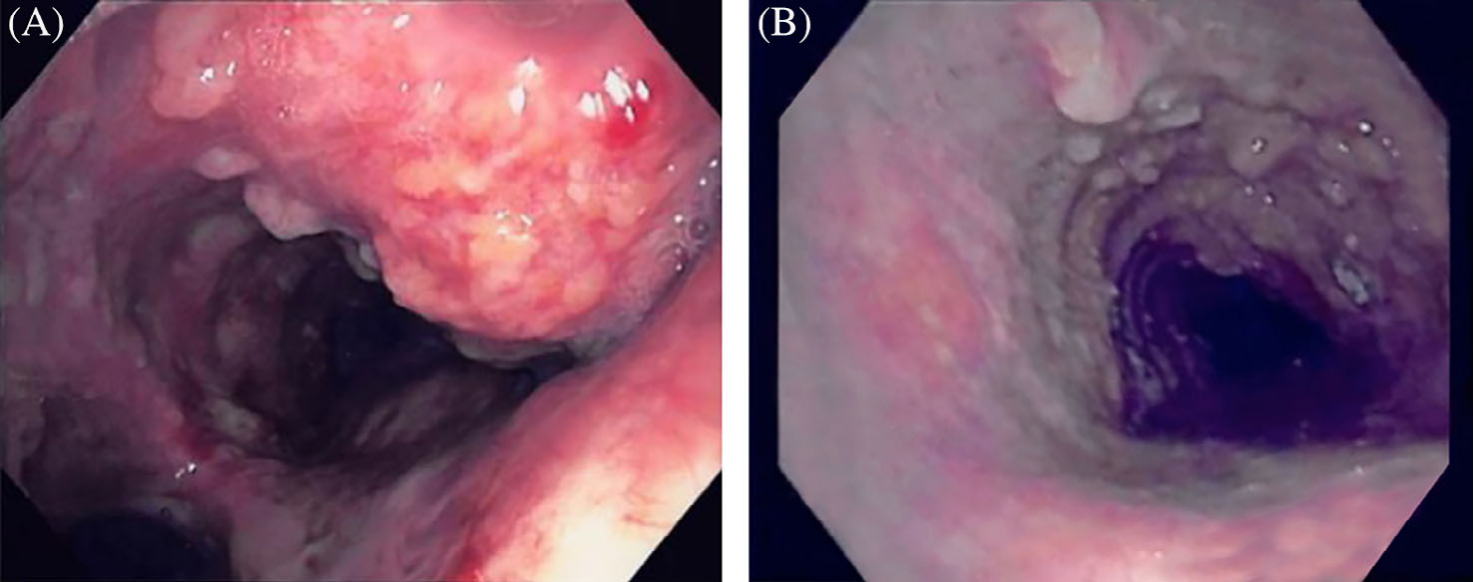
(A) Protruding nodularity at the level of carina. (B) Tracheal wall with widespread nodularity, consistent with tracheobronchopathia osteochondroplastica.

**Figure 3:**
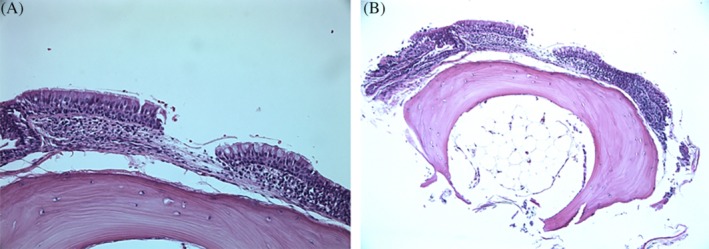
Histopathological examination showed ciliated respiratory mucosa with foci of metaplastic bone within stroma.

Twelve months later, the patient presented for a preoperative assessment prior to elective spinal surgery. Repeat bronchoscopy revealed narrowing of right upper lobe bronchus but without evidence of critical stenosis in the subsegments. These findings were similar to those from 12 months prior and he underwent spinal surgery without any perioperative complications (Figs. 1, 2, 3).

## Discussion

TO is a benign chronic disease characterized by the presence of submucosal osteocartilaginous nodules in the tracheobronchial tree with unknown aetiology 6.

Most patients with TO are asymptomatic. Some report non‐specific respiratory symptoms including chronic cough, dyspnoea, recurrent pneumonia, or haemoptysis. Occasionally, unexpected difficult intubation might be the first manifestation of the disease 4.

Diagnosis is based on computed tomography (CT) findings and typical bronchoscopic manifestations with/without histopathological examination. Histopathology, although not necessary, is useful in excluding other differential diagnoses including papillomatoses, amyloidosis, sarcoidosis, chondrosarcoma, dermatomyositis, scleroderma, hamartomas, relapsing polychondritis, granulomatosis with polyangiitis, and malignancy 3, 5.

Conservative management is appropriate in asymptomatic patients as lesions grow slowly. If symptomatic, endoscopic treatments including Nd:YAG laser photo evaporation, coring of the lesions with rigid bronchoscope, and endobronchial stent placement can be used 7.

Avoidance of airway manipulation is ideal in patients with known TO during operative intervention. If this is inevitable, laryngeal mask airway has been shown to be useful. It has been used successfully in one patient with TO requiring general anaesthesia for an intra‐abdominal procedure 8. With large subglottic TO lesions, an awake fibreoptic intubation may be the preferred airway management technique.

In elective procedures, patients should undergo detailed preoperative evaluation including fibreoptic bronchoscopy to assess patency of the airway, estimate ETT size, and if necessary surgical or bronchoscopic procedure to rectify stenosis 9.

In summary, although rare, TO is one of the causes of difficult intubation. We think comprehensive assessment, including fibreoptic bronchoscopy, prior to elective surgical procedures is important to evaluate airway patency and estimate the size of endobronchial tube. Prior to and following extubation, the patient should be monitored for possible airway oedema and haemorrhage.

### Disclosure Statement

Appropriate written informed consent was obtained for publication of this case report and accompanying images.
